# 2,2′-[Naphthalene-1,5-diylbis(nitrilo­methanylyl­idene)]diphenol

**DOI:** 10.1107/S1600536811023099

**Published:** 2011-06-22

**Authors:** S. Karthikeyan, J. Muthukumaran, R. Krishna, Bala. Manimaran

**Affiliations:** a Department of Chemistry, Pondicherry University, Puducherry 605 014, India; b Centre for Bioinformatics, Pondicherry University, Puducherry 605 014, India

## Abstract

The title compound, C_24_H_18_N_2_O_2_, lies about an inversion centre and the asymmetric unit contains one half-mol­ecule. An intra­molecular O—H⋯N hydrogen bond generates a six-membered ring, producing an *S*(6) ring motif. The crystal packing exhibits inter­molecular π–π stacking inter­actions between the aromatic rings with a centroid–centroid distance of 3.851 (2) Å.

## Related literature

For hydrogen-bond motifs, see: Bernstein *et al.* (1995 ▶). For applications of Schiff base ligands, see: Pandeya *et al.* (1999 ▶, 2000 ▶); Singh & Dash (1988 ▶); Kelley *et al.* (1995 ▶); Turan-Zitouni *et al.* (2007 ▶); Tarafder *et al.* (2002 ▶); Sakyan *et al.* (2004 ▶); Gianneshi *et al.* (2005 ▶); Morris *et al.* (2001 ▶); Lu *et al.* (2007 ▶); Lau *et al.* (1999 ▶). For a related structure, see: Al-Douh *et al.* (2009 ▶).
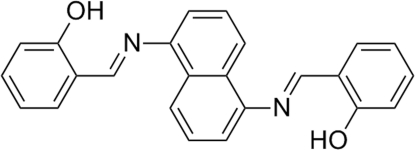


## Experimental

### 

#### Crystal data

C_24_H_18_N_2_O_2_
*M* *_r_* = 366.40
Monoclinic, 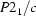

*a* = 3.8510 (9) Å
*b* = 19.395 (6) Å
*c* = 11.796 (2) Å
β = 95.85 (3)°
*V* = 876.4 (4) Å^3^
*Z* = 2
Mo *K*α radiation
μ = 0.09 mm^−1^
*T* = 293 K
0.23 × 0.15 × 0.11 mm

#### Data collection

Oxford Diffraction Xcalibur-S diffractometer
Absorption correction: multi-scan (*CrysAlis PRO*; Oxford Diffraction, 2009 ▶) *T*_min_ = 0.980, *T*_max_ = 0.990
4711 measured reflections
1544 independent reflections
1004 reflections with *I* > 2σ(*I*)
*R*_int_ = 0.099

#### Refinement

*R*[*F* ^2^ > 2σ(*F* ^2^)] = 0.080
*wR*(*F* ^2^) = 0.206
*S* = 1.18
1544 reflections
128 parameters
H-atom parameters constrained
Δρ_max_ = 0.35 e Å^−3^
Δρ_min_ = −0.36 e Å^−3^

### 

Data collection: *CrysAlis PRO* (Oxford Diffraction, 2009 ▶); cell refinement: *CrysAlis PRO*; data reduction: *CrysAlis PRO*; program(s) used to solve structure: *SHELXS97* (Sheldrick, 2008 ▶); program(s) used to refine structure: *SHELXL97* (Sheldrick, 2008 ▶); molecular graphics: *ORTEP-3 for Windows* (Farrugia, 1997 ▶); software used to prepare material for publication: *PLATON* (Spek, 2009 ▶).

## Supplementary Material

Crystal structure: contains datablock(s) I, global. DOI: 10.1107/S1600536811023099/sj5163sup1.cif

Structure factors: contains datablock(s) I. DOI: 10.1107/S1600536811023099/sj5163Isup2.hkl

Additional supplementary materials:  crystallographic information; 3D view; checkCIF report

## Figures and Tables

**Table 1:** Hydrogen-bond geometry (Å, °)

*D*—H⋯*A*	*D*—H	H⋯*A*	*D*⋯*A*	*D*—H⋯*A*
O1—H1⋯N1	0.82	1.90	2.634 (3)	148

**Fig. 1.**
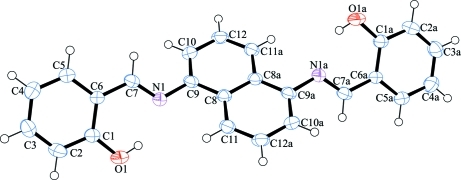
The molecular structure of title compound, showing displacement ellipsoids drawn at the 50% probability level.

**Fig. 2.**
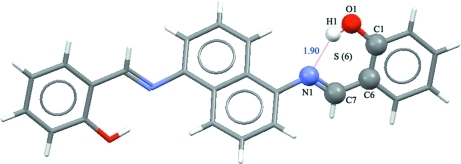
A view of the intramolecular S (6) ring motif formed by an O—H···N interaction in the title compound. The motif forming atoms are shown in a ball and stick representation and the hydrogen bond is shown as a red dashed line.

**Fig. 3.**
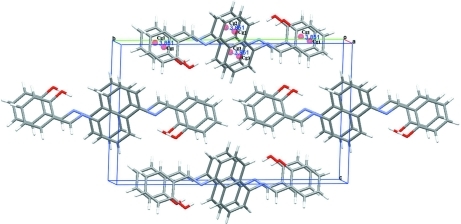
View of the crystal packing showing intermolecular π···π stacking interactions. Cg(1), Cg(2) and Cg(3) are the centroids of the C1/C2/C3/C4/C5/C6, C8/C9/C10/C12/C11a/C8a and C8/C11/C12a/C10a/C9a/C8a rings respectively.

## supplementary crystallographic information

### Comment

Schiff base ligands exhibit several biological and pharmacological properties such as anti-viral, anti-cancer, anti-bacterial, anti-fungal, anti-inflammatory, anti-convulsant and anti-HIV activities (Pandeya *et al.*, 1999, 2000; Singh & Dash, 1988; Kelley *et al.*, 1995; Turan-Zitouni *et al.*, 2007; Tarafder *et al.*, 2002). They are well known to be medicinally important and have been used to design several medicinal compounds (Sakyan *et al.*, 2004). Schiff base ligands have been extensively employed in various fields such as catalysis (Gianneshi *et al.*, 2005), materials chemistry (Morris *et al.*, 2001) and magneto chemistry (Lu *et al.*, 2007). Schiff bases that incorporate an imine group (–CH=N), are used in elucidating the transformation and rasemination mechanism in biological systems (Lau *et al.*, 1999). In view of the growing medicinal importance of Schiff base ligands and their derivatives, a single-crystal X-ray diffraction study on the title compound was carried out and analyzed. The molecular structure of the title compound is shown in Fig. 1. The bond length of imine group (–CH=N) is comparable to those observed in a related crystal structure namely that of 6,6'-Dimethoxy-2,2'-[*p*-phenylene-bis(nitrilomethylidyne)]diphenol chloroform disolvate (Al-Douh *et al.*, 2009). The atom series and their weighted average absolute torsion angles in all six-membered ring of title compound are Ring 1: C1/C2/C3/C4/C5/C6 & 0.2°, Ring 2: C8/C9/C10/C12/C11*a*/C8a & 0.8° and Ring 3: C8/C11/C12*a*/C10*a*/C9a/C8a & 0.8°. The molecular structure is stabilized by intramolecular O1—H1···N1 interactions. An intramolecular O1—H1···N1 hydrogen bond generates a six-membered ring, producing an *S*(6) ring motif [Fig. 2] [O1—H1 distance: 0.82 Å, H1—N1 distance: 1.90 Å, O1—N1 distance: 2.634 (3) Å and O1—H1···N1 angle 148 °]. (Bernstein *et al.*, 1995). The crystal packing exhibits intermolecular π—π stacking interactions (Fig. 3) between the aromatic rings with the centroid-to-centroid distance of 3.851 (2) Å.

### Experimental

1,5-naphthalenediamine (158 mg, 1 mmol) was taken in a 100 ml round bottom schlenk flask and the system was evacuated and purged with nitrogen. To this, a freshly distilled ethanol (40 ml), salicylaldehyde (0.2 ml, 2 mmol) and 2 drops of acetic acid were added. The reaction mixture was stirred at 25 °C for 3 h. The solvent was evaporated using vacuum and the yellow color product was purified by recrystallization with dichloromethane (Yield 73%, Melting Point: 220 °C). Single crystals suitable for X-ray diffraction were prepared by slow evaporation of a solution of the title compound in dichloromethane at room temperature. Spectroscopic data of the title compound: IR (KBr): ν 3442 (*m*), 1615 (*s*), 1454 (w), 1278 (*m*), 1143 (*m*), 744 (*s*), cm^-1. 1^H NMR (400 MHz, CDCl_3_): δ 13.30 (s, 2H), 8.74 (s, 2H), 8.21 (d, 2H), 7.57 (t, 2H), 7.49–7.72 (m, 4H), 7.25 (d, 2H), 7.12 (d, 2H), 7.0 (t, 2H). ^13^C NMR (100 MHz, CDCl_3_): δ 164.0, 161.4, 146.4, 133.7, 132.6, 129.0, 126.7, 122.4, 119.6, 119.4, 117.5, 115.0.

### Refinement

The non-hydrogen atoms were refined anisotropically whereas hydrogen atoms were refined isotropically. The hydrogen atoms were placed in calculated positions (C–H = 0.93 Å) and included in the refinement in a riding-model approximation with *U*_iso_(H) = 1.2Ueq(C).

### Crystal data

**Table:**

C_24_H_18_N_2_O_2_	*F*(000) = 384
*M**_r_* = 366.40	*D*_x_ = 1.388 Mg m^−^^3^
Monoclinic, *P*2_1_/*c*	Mo *K*α radiation, λ = 0.71073 Å
Hall symbol: -P 2ybc	Cell parameters from 4711 reflections
*a* = 3.8510 (9) Å	θ = 3.5–25.0°
*b* = 19.395 (6) Å	µ = 0.09 mm^−^^1^
*c* = 11.796 (2) Å	*T* = 293 K
β = 95.85 (3)°	Block, yellow
*V* = 876.4 (4) Å^3^	0.23 × 0.15 × 0.11 mm
*Z* = 2	

### Data collection

**Table:**

Oxford Diffraction Xcalibur-S diffractometer	1544 independent reflections
Radiation source: fine-focus sealed tube	1004 reflections with *I* > 2σ(*I*)
graphite	*R*_int_ = 0.099
Detector resolution: 15.9948 pixels mm^-1^	θ_max_ = 25.0°, θ_min_ = 3.5°
ω scans	*h* = −4→3
Absorption correction: multi-scan (*CrysAlis PRO*; Oxford Diffraction, 2009)	*k* = −22→23
*T*_min_ = 0.980, *T*_max_ = 0.990	*l* = −13→13
4711 measured reflections	

### Refinement

**Table:**

Refinement on *F*^2^	Primary atom site location: structure-invariant direct methods
Least-squares matrix: full	Secondary atom site location: difference Fourier map
*R*[*F*^2^ > 2σ(*F*^2^)] = 0.080	Hydrogen site location: inferred from neighbouring sites
*wR*(*F*^2^) = 0.206	H-atom parameters constrained
*S* = 1.18	*w* = 1/[σ^2^(*F*_o_^2^) + (0.1*P*)^2^] where *P* = (*F*_o_^2^ + 2*F*_c_^2^)/3
1544 reflections	(Δ/σ)_max_ < 0.001
128 parameters	Δρ_max_ = 0.35 e Å^−^^3^
0 restraints	Δρ_min_ = −0.36 e Å^−^^3^

### Special details

**Table:**

Geometry. All e.s.d.'s (except the e.s.d. in the dihedral angle between two l.s. planes) are estimated using the full covariance matrix. The cell e.s.d.'s are taken into account individually in the estimation of e.s.d.'s in distances, angles and torsion angles; correlations between e.s.d.'s in cell parameters are only used when they are defined by crystal symmetry. An approximate (isotropic) treatment of cell e.s.d.'s is used for estimating e.s.d.'s involving l.s. planes.
Refinement. Refinement of *F*^2^ against ALL reflections. The weighted *R*-factor *wR* and goodness of fit *S* are based on *F*^2^, conventional *R*-factors *R* are based on *F*, with *F* set to zero for negative *F*^2^. The threshold expression of *F*^2^ > σ(*F*^2^) is used only for calculating *R*-factors(gt) *etc*. and is not relevant to the choice of reflections for refinement. *R*-factors based on *F*^2^ are statistically about twice as large as those based on *F*, and *R*- factors based on ALL data will be even larger.

### Fractional atomic coordinates and isotropic or equivalent isotropic displacement parameters (Å^2^)

**Table:**

	*x*	*y*	*z*	*U*_iso_*/*U*_eq_	
N1	0.3017 (6)	0.64602 (13)	−0.0063 (2)	0.0372 (7)	
C1	0.5621 (8)	0.77817 (16)	0.0770 (3)	0.0380 (8)	
C6	0.3700 (7)	0.76789 (15)	−0.0294 (3)	0.0355 (8)	
C8	0.0499 (7)	0.53316 (14)	0.0215 (3)	0.0331 (8)	
C7	0.2450 (8)	0.70066 (16)	−0.0666 (3)	0.0390 (8)	
H7	0.1174	0.6968	−0.1376	0.047*	
C9	0.1929 (7)	0.58080 (15)	−0.0513 (3)	0.0361 (8)	
O1	0.6420 (7)	0.72510 (12)	0.1490 (2)	0.0531 (8)	
H1	0.5617	0.6893	0.1202	0.080*	
C11	−0.0030 (8)	0.54993 (16)	0.1351 (3)	0.0381 (8)	
H11	0.0586	0.5934	0.1638	0.046*	
C5	0.2987 (9)	0.82501 (16)	−0.1005 (3)	0.0428 (9)	
H5	0.1709	0.8190	−0.1711	0.051*	
C10	0.2399 (8)	0.56269 (17)	−0.1607 (3)	0.0384 (8)	
H10	0.3368	0.5942	−0.2078	0.046*	
C2	0.6767 (8)	0.84352 (17)	0.1085 (3)	0.0442 (9)	
H2	0.8043	0.8504	0.1788	0.053*	
C12	−0.1435 (8)	0.50288 (16)	0.2025 (3)	0.0398 (8)	
H12	−0.1760	0.5143	0.2773	0.048*	
C3	0.6030 (9)	0.89849 (18)	0.0363 (3)	0.0484 (10)	
H3	0.6824	0.9422	0.0585	0.058*	
C4	0.4136 (9)	0.88975 (18)	−0.0682 (3)	0.0471 (9)	
H4	0.3641	0.9273	−0.1163	0.057*	

### Atomic displacement parameters (Å^2^)

**Table:**

	*U*^11^	*U*^22^	*U*^33^	*U*^12^	*U*^13^	*U*^23^
N1	0.0418 (13)	0.0354 (15)	0.0331 (17)	−0.0021 (11)	−0.0027 (12)	0.0029 (12)
C1	0.0390 (15)	0.0432 (19)	0.031 (2)	0.0014 (13)	0.0018 (15)	0.0017 (15)
C6	0.0381 (15)	0.0395 (18)	0.028 (2)	−0.0038 (13)	−0.0007 (14)	0.0010 (15)
C8	0.0324 (13)	0.0363 (16)	0.0288 (19)	0.0033 (12)	−0.0058 (13)	0.0026 (14)
C7	0.0399 (16)	0.0445 (19)	0.031 (2)	−0.0027 (13)	−0.0045 (14)	−0.0004 (15)
C9	0.0338 (14)	0.0405 (18)	0.032 (2)	−0.0013 (13)	−0.0065 (14)	−0.0012 (15)
O1	0.0687 (16)	0.0534 (15)	0.0332 (16)	−0.0045 (12)	−0.0141 (12)	0.0057 (12)
C11	0.0404 (16)	0.0384 (17)	0.034 (2)	0.0013 (13)	−0.0035 (14)	−0.0021 (16)
C5	0.0516 (18)	0.0440 (19)	0.031 (2)	−0.0015 (15)	−0.0023 (16)	0.0016 (16)
C10	0.0400 (16)	0.0456 (19)	0.029 (2)	−0.0017 (13)	0.0012 (14)	0.0067 (15)
C2	0.0442 (17)	0.052 (2)	0.034 (2)	−0.0037 (15)	−0.0051 (15)	−0.0082 (16)
C12	0.0443 (16)	0.051 (2)	0.0241 (19)	0.0029 (14)	0.0012 (15)	−0.0032 (15)
C3	0.0515 (19)	0.0410 (19)	0.052 (3)	−0.0047 (15)	0.0024 (18)	−0.0059 (18)
C4	0.056 (2)	0.043 (2)	0.042 (2)	0.0001 (15)	0.0030 (18)	0.0070 (17)

### Geometric parameters (Å, °)

**Table:**

N1—C7	1.283 (4)	C11—C12	1.358 (5)
N1—C9	1.418 (4)	C11—H11	0.9300
C1—O1	1.350 (4)	C5—C4	1.372 (5)
C1—C2	1.381 (4)	C5—H5	0.9300
C1—C6	1.404 (4)	C10—C12^i^	1.400 (4)
C6—C5	1.400 (4)	C10—H10	0.9300
C6—C7	1.443 (4)	C2—C3	1.376 (5)
C8—C9	1.411 (5)	C2—H2	0.9300
C8—C11	1.414 (4)	C12—C10^i^	1.400 (4)
C8—C8^i^	1.421 (6)	C12—H12	0.9300
C7—H7	0.9300	C3—C4	1.378 (5)
C9—C10	1.367 (5)	C3—H3	0.9300
O1—H1	0.8200	C4—H4	0.9300
			
C7—N1—C9	120.2 (3)	C8—C11—H11	119.8
O1—C1—C2	119.0 (3)	C4—C5—C6	121.3 (3)
O1—C1—C6	121.3 (3)	C4—C5—H5	119.3
C2—C1—C6	119.7 (3)	C6—C5—H5	119.3
C1—C6—C5	118.4 (3)	C9—C10—C12^i^	120.7 (3)
C1—C6—C7	122.0 (3)	C9—C10—H10	119.7
C5—C6—C7	119.6 (3)	C12^i^—C10—H10	119.7
C9—C8—C11	121.9 (3)	C3—C2—C1	120.3 (3)
C9—C8—C8^i^	119.0 (4)	C3—C2—H2	119.8
C11—C8—C8^i^	119.1 (4)	C1—C2—H2	119.8
N1—C7—C6	123.0 (3)	C11—C12—C10^i^	120.7 (3)
N1—C7—H7	118.5	C11—C12—H12	119.6
C6—C7—H7	118.5	C10^i^—C12—H12	119.6
C10—C9—C8	120.2 (3)	C2—C3—C4	121.0 (3)
C10—C9—N1	121.3 (3)	C2—C3—H3	119.5
C8—C9—N1	118.4 (3)	C4—C3—H3	119.5
C1—O1—H1	109.5	C5—C4—C3	119.2 (3)
C12—C11—C8	120.3 (3)	C5—C4—H4	120.4
C12—C11—H11	119.8	C3—C4—H4	120.4
			
O1—C1—C6—C5	−179.4 (3)	C9—C8—C11—C12	179.6 (3)
C2—C1—C6—C5	−0.3 (5)	C8^i^—C8—C11—C12	0.8 (5)
O1—C1—C6—C7	0.7 (5)	C1—C6—C5—C4	0.3 (5)
C2—C1—C6—C7	179.8 (3)	C7—C6—C5—C4	−179.9 (3)
C9—N1—C7—C6	−175.5 (3)	C8—C9—C10—C12^i^	0.7 (4)
C1—C6—C7—N1	−0.5 (5)	N1—C9—C10—C12^i^	177.5 (3)
C5—C6—C7—N1	179.7 (3)	O1—C1—C2—C3	179.2 (3)
C11—C8—C9—C10	−180.0 (3)	C6—C1—C2—C3	0.1 (5)
C8^i^—C8—C9—C10	−1.2 (5)	C8—C11—C12—C10^i^	−0.4 (4)
C11—C8—C9—N1	3.2 (4)	C1—C2—C3—C4	0.2 (5)
C8^i^—C8—C9—N1	−178.0 (3)	C6—C5—C4—C3	0.1 (5)
C7—N1—C9—C10	43.0 (4)	C2—C3—C4—C5	−0.3 (5)
C7—N1—C9—C8	−140.2 (3)		

Symmetry codes: (i) −*x*, −*y*+1, −*z*.

### Hydrogen-bond geometry (Å, °)

**Table:**

*D*—H···*A*	*D*—H	H···*A*	*D*···*A*	*D*—H···*A*
O1—H1···N1	0.82	1.90	2.634 (3)	148
